# Effects of Clobetasol in an Aging Mouse Model of Spinal Cord Hemisection

**DOI:** 10.3390/biology14111595

**Published:** 2025-11-14

**Authors:** Maria Ciuro, Maria Sangiorgio, Giuliano Cantone, Carlo Fichera, Valeria Cacciato, Giampiero Leanza, Rosario Gulino

**Affiliations:** 1 Department of Biomedical and Biotechnological Sciences, University of Catania, Via Santa Sofia 89, 95123 Catania, Italy; ciuromaria@hotmail.it (M.C.); ma.sangiorgio@outlook.it (M.S.); cantonegiuliano@hotmail.com (G.C.); carl.fichera@gmail.com (C.F.); valeria.cacciato@gmail.com (V.C.); 2 Department of Drug and Health Sciences, University of Catania, Via Santa Sofia 64, 95125 Catania, Italy; gpleanza@unict.it

**Keywords:** aging, clobetasol, hemisection, mouse, plasticity, sonic hedgehog, spinal cord injury, physiology

## Abstract

Spinal cord injury leads to permanent neurological deficits, and aging further diminishes the plasticity and regenerative responses required for recovery. Activation of the Sonic hedgehog (Shh) pathway through the receptor Smoothened (Smo) has been proposed as a potential strategy to promote repair, and clobetasol, a potent glucocorticoid, has been identified as a pharmacological Smo agonist. However, the possible restorative effect of Smo agonists has never been studied during aging. Here, the effects of clobetasol treatment have been investigated in aging mice following spinal cord hemisection. Animals received weekly systemic injections of clobetasol or vehicle and were monitored for 11 weeks using the Basso Mouse Scale and open field test, followed by post-mortem histological analysis. Vehicle-treated mice exhibited a modest spontaneous recovery of locomotor function, whereas clobetasol-treated mice failed to improve and displayed significantly worse motor performance. Histological evaluation revealed reduced synaptic density in clobetasol-treated mice. Moreover, microglia/macrophage reaction was increased in vehicle-treated injured mice but suppressed by clobetasol, consistent with glucocorticoid-mediated inhibition of inflammatory responses. Together, these findings indicate that in aged animals clobetasol administration does not enhance plasticity or promote recovery but instead exacerbates synaptic loss and functional deficits. These results underscore the importance of age as a determinant of therapeutic efficacy after spinal cord injury.

## Simple Summary

Spinal cord injury causes permanent damage to the nerves that control sensory and motor functions, leaving people with severe disability. Recovery after such an injury is very limited, and this problem becomes even worse with aging, when the brain and spinal cord lose much of their ability to repair themselves. Scientists have been testing drugs that might boost the natural capacity of nerve cells to grow and make new connections. One such drug is clobetasol, a strong anti-inflammatory steroid that has also been suggested to activate a pathway important for nerve repair. In this study, clobetasol has been tested in old mice with a spinal cord injury. The results show that animals treated with clobetasol did not recover better than untreated animals. In fact, they performed worse in movement tests. Examination of the spinal cord tissue showed fewer nerve connections in the treated animals, while the usual immune response to injury was strongly reduced. These results suggest that, in older individuals, clobetasol may suppress helpful processes that support recovery, leading to worse outcomes. These findings underline the importance of including the effects of aging in the research for potential treatments of spinal cord injury.

## 1. Introduction

Spinal cord injury (SCI) is one of the most catastrophic injuries to the central nervous system (CNS), causing permanent damage to motor and sensory pathways and resulting in long-lasting functional deficits [1,2,3,4]. After SCI, tissue damage is worsened by secondary processes such as inflammation, excitotoxicity, glial scar formation, and accumulation of inhibitory molecules, all of which further restrict recovery [3,5,6].

A large body of evidence has so far proven that sensory–motor recovery is possible in adult mammals, as a result of various forms of spontaneous and activity-dependent neuroplasticity occurring in spared descending pathways, sensory afferents, and spinal central pattern generators, which can be collectively named as spinal learning [7,8,9,10,11,12].

However, both the amount of tissue damage and the capacity for plastic changes after CNS injury can be affected by aging in both human patients and animal models [13,14,15,16,17,18,19]. For example, it is known that PTEN (Phosphatase and TENsin homolog) is a negative regulator of the mTOR (mammalian target of rapamycin) signaling pathway and that neuronal mTOR activity declines with aging and is further decreased after injury [20,21,22]. Deletion of PTEN in adult mice enhances corticospinal tract axonal regeneration and motor recovery after SCI, indicating that intrinsic growth pathways can be reactivated in adulthood. However, the same regenerative manipulations generally yield smaller effects, or even fail when applied in older animals, and this is accompanied by a downregulation of mTOR activity, suggesting that both cellular responsiveness and microenvironmental permissiveness factors degrade with age [20,21,22].

At the molecular level, the Sonic Hedgehog (Shh) signaling pathway plays an essential role as a regulator of neuroprotection, neuroinflammation, and plasticity [23,24,25]. Shh is a morphogen regulating the neural patterning during development [26] as well as the self-renewal and differentiation of neural precursor cells in the adult CNS [27,28]. The secreted Shh ligand binds to its receptor Patched that activates the Smoothened (Smo) receptor, which ultimately induces the translocation of the transcription factors Gli1-3 to the nucleus, where they regulate the expression of target genes [29].

Several experimental studies have highlighted the potential of Shh signaling as a therapeutic target for SCI. Pharmacological or genetic activation of the pathway has been shown to stimulate neural progenitor proliferation, enhance oligodendrocyte differentiation, reduce apoptosis, and improve functional outcomes in rodent models [30,31,32,33]. Although most of these studies have been performed in young adult rodents, the evidence suggests that Shh activation might counteract age-related declines in neuroplasticity and regenerative potential, raising the possibility that pharmacological stimulation of this pathway could mitigate the detrimental impact of aging on recovery after spinal cord injury. In fact, recent studies have shown that an intact Shh/Smo signaling is not only required after injury but also throughout aging to maintain restorative capacity [34,35]. Therefore, Shh signaling represents a druggable target potentially capable of promoting functional recovery after injury [36,37,38].

Clobetasol, an already approved synthetic glucocorticoid, has been identified as a Smo agonist and as a promoter of remyelination, neuroprotection, and repair [39,40,41]. However, in these studies, the restorative actions of clobetasol, or other Smo agonists, have consistently been investigated in young recipients displaying full capacity for efficient compensatory responses. By contrast, no data are presently available about its efficacy in aged subjects, where such capacity is somewhat reduced or compromised.

Given these intersecting lines of evidence, it would be of interest to verify if clobetasol treatment in aging animals after SCI can support functional recovery. The present study aims to do this by conducting longitudinal behavioral assessments in aged mice subjected to spinal cord hemisection, comparing motor recovery under clobetasol vs. vehicle treatment. Two complementary behavioral assays—the Basso Mouse Scale (BMS) and open field locomotion—were used to detect locomotor ability. By measuring and comparing recovery over 11 weeks after lesion, this work aimed to clarify how aging and clobetasol interact to influence plasticity, regeneration, and functional outcome after SCI.

## 2. Materials and Methods

### 2.1. Mouse Model of Spinal Cord Hemisection

All experiments were performed in accordance with the European and Italian regulations (2010/63/EU and Italian D. Lgs. no. 26/2014) and following the ARRIVE guidelines. All efforts were made to replace, reduce, and refine the use of laboratory animals. Moreover, the study was conducted in accordance with the recommendations of the local committee for animal welfare (OPBA, University of Catania, Catania, Italy); the protocol was approved by OPBA and by the Italian Ministry of Health (protocol no. 477/2019-PR).

Twenty-three male mice (strain: C57BL/6N) from Charles River Laboratories Italia s.r.l. (Calco, Italy), 12 months old, were used for this experiment. Animals were randomly assigned to different cages (n ≤ 5 animals per cage) and kept under constant temperature (23–25 °C) with ad libitum access to food and water.

For spinal cord hemisection, mice were anesthetized with isoflurane (4% for induction and 2% for maintenance), their column was stabilized with a vertebral clamp, and a hole was drilled on the dorsal surface of the ninth thoracic vertebra (corresponding to the spinal segments T10–T11). Then, after removal of the dura mater, the right side of the cord was transected by means of a microsurgical knife attached to an arm of the stereotaxic device, under visual guidance using an operating microscope. Care was taken in order to avoid damage to the median blood vessels of the spinal cord. The lesion site was filled with sterile gelfoam, and muscle and skin were then sutured. After the injury, mice were placed one per cage and randomly assigned to the treatment groups (by a random number generator).

**Figure 1:**
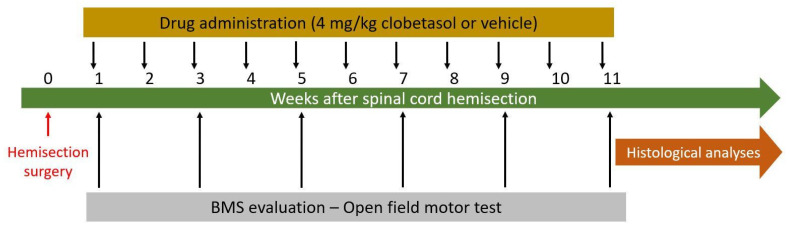
Schematic representation of the experimental timeline.

The experimental workflow is summarized in Figure 1. In particular, subgroups of hemisected mice received an intraperitoneal injection of either clobetasol propionate (4 mg/kg; Hem-Clob; n = 9) or vehicle alone (dimethyl sulfoxide; Hem-Veh; n = 9) 3 days post lesion (dpl). This dosing regimen has been previously used in another study carried out in our laboratory, where it proved to be both effective and non-toxic [40]. Other researchers have administered the drug daily at a dose of 2 mg/kg, mainly in demyelination models [39,41]. However, several studies have also demonstrated that intermittent glucocorticoid dosing can promote regenerative effects while limiting side effects compared with daily administration [42,43]. Treatments were then repeated once a week at 6, 13, 20, 27, 34, 41, 48, 55, 62, 69, and 76 dpl, and motor activity was evaluated during this period by BMS [44] and open field (please see below).

A small group of age-matched mice without any surgical intervention or treatment was included as a control group (intact; n = 5).

A power analysis was performed using the Systat software package, version 11 (Systat Software, Inc., Chicago, IL, USA), to determine the appropriate group size. The parameters used for this analysis were derived from previous studies conducted in our laboratory that also employed the hemisection model [8].

### 2.2. Evaluating the Spinal Cord Injury by BMS

The locomotory behavior of mice was tested and scored according to guidelines of BMS [44]. BMS tests were performed at 1, 3, 5, 7, 9, and 11 weeks after hemisection. Each animal was individually placed on a flat, non-slip surface, allowed to move freely, and observed for 5 min by two independent evaluators, blind to the drug treatments (cages were codified). Scoring was based on different parameters such as ankle movements, paw placement, coordination, trunk instability, and tail position (please see Table 1), with a minimum score of 0 (no movement) to a maximum score of 9 (normal locomotion).

### 2.3. Evaluating the Locomotor Activity by Open Field

After acclimation to the behavioral device, at 1, 3, 5, 7, 9, and 11 weeks after the lesion, animals were placed individually in a 40 × 40 cm open-field arena and allowed to move freely and explore for 4 min. Motor performance of each animal was recorded by a tracking camera placed above the open-field arena, and the videos were analyzed thereafter to evaluate the following parameters: distance (i.e., the total length of path in 4 min); speed (i.e., the mean instantaneous speed calculated among the bouts of locomotion with velocity > 30 mm/s); number of bouts of locomotion; acceleration (i.e., the mean value of positive and/or negative accelerations calculated among the bouts of locomotion with velocity > 30 mm/s). Investigators were blind to the drug treatments (cages were codified).

### 2.4. Histology and Immunofluorescence

At the end of the survival period (77 dpl), all animals were sacrificed by intracardial perfusion, and the spinal cords were dissected out, post-fixed for 24 h in 4% paraformaldehyde in 0.1 M phosphate buffer, and cryoprotected by immersion in 20% sucrose. Spinal cords were embedded in Optimum Cutting Temperature (OCT) and sectioned using a freezing microtome. In order to study tissue pathology, immunofluorescence was performed.

For immunofluorescence staining, sections were incubated with 5% normal donkey serum and 0.4% Triton in PBS for 1 h and then overnight at room temperature with an appropriate combination of one of the following antibodies diluted in 1% normal donkey serum in PBS and 0.3% Triton: mouse monoclonal anti-Gli1 (Proteintech, Cat. no.: 66905-1, 1:100, Rosemont, IL, USA); rabbit polyclonal anti-synaptophysin (Santa Cruz Biotechnology, Cat. no.: sc9116, 1:70, Dallas, TX, USA); goat polyclonal anti-choline acetyl transferase (ChAT; Chemicon, Cat. no.: AB144P, 1:150); goat polyclonal anti-Iba1 (Novus Biologicals, Cat. no.: NB100-1028, 1:100, Centennial, CO, USA). Samples were then washed and incubated for 1 h at room temperature with the appropriate secondary antibody diluted 1:500 in 1% normal donkey serum in PBS and 0.3% Triton: Alexa Fluor 546 donkey anti-goat (Thermo Fisher Scientific, Cat. no.: A-11056, Waltham, MA, USA); Alexa Fluor 546 donkey anti-mouse (Thermo Fisher Scientific, Cat. no.: A-10036, Waltham, MA, USA); Alexa Fluor 488 donkey anti-rabbit (Thermo Fisher Scientific, Cat. no.: A-11008, Waltham, MA, USA). Nuclei were counterstained with DAPI 1:1000 in PBS. Slides were coverslipped with BrightMount (Abcam, Cat. no.: ab103746, Cambridge, UK). For quantification of Gli1, synaptophysin and Iba1 staining intensity, as well as for colocalization assessment, n ≥ 10 regions of interest (photos taken from the Rexed lamina IX of the lumbar spinal cord, below the level of the lesion) per n = 3 sections per animal were analyzed using a fluorescence microscope (Nikon Eclipse 80i; Nikon Europe B.V., Amstelveen, The Netherlands) and photos were analyzed by investigators blind to the treatment, using ImageJ software, version 1.53e. In particular, synaptic puncta in the lamina IX of the spinal cord were quantified by measuring the average optical density, and this parameter can account for the number of synaptic densities within the circuitries surrounding the motoneuron bodies in the lumbar spinal cord [40]. Similarly, the Iba1 optical density in the lumbar spinal cord has been employed as a measure of microglia/macrophage response, as a result of spinal cord injury and drug treatment [45].

### 2.5. Statistical Analysis

Group size has been determined by power analysis by using BMS score as the outcome measure. Data were tested for normality using a D’Agostino and Pearson omnibus normality test and subsequently assessed for homogeneity of variance. Then data were analyzed as mean values ± SEM (standard error of the mean) and normalized to control values, where appropriate. Differences among experimental groups were evaluated by using Mixed-design ANOVA or One-way ANOVA followed by Fisher’s Least-Significant-Difference post hoc test, where appropriate. For all experiments, a *p*-value of <0.05 was considered to be significant. All analyses were performed by means of Systat package version 11 (Systat Software, Inc., Chicago, IL, USA).

## 3. Results

All animals survived the surgical procedure and the subsequent experimental treatments, with the exception of one mouse belonging to the Hem-Clob group, which died during the course of the study. The remaining animals were carefully monitored throughout the experimental timeline, and no major complications related to anesthesia, surgery, or handling were recorded.

**Figure 2:**
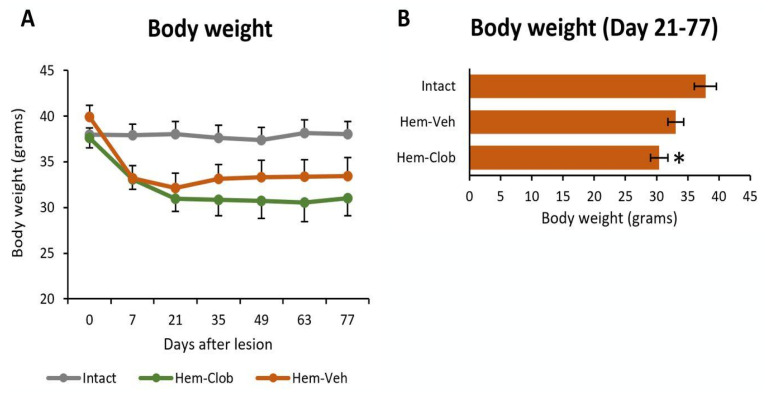
Body weight changes after spinal cord hemisection and drug treatments. (**A**) Timeline of body weight measurements from surgery to animal sacrifice. (**B**) Comparison of the average body weight of animals belonging to the different experimental groups. The asterisk indicates significant difference (*p* < 0.05) from the Intact group.

As expected, spinal cord hemisection induced a transient weight loss in the injured mice. Animals in the Hem-Veh group showed a moderate decrease in body weight during the first three weeks after surgery, which stabilized thereafter (Figure 2A). Their body weights remained slightly lower than presurgical values but showed no further decline over the following weeks (Figure 2A), and the difference from intact controls did not reach statistical significance (One-way ANOVA followed by Fisher’s LSD post hoc test, *p* > 0.05, Figure 2B).

In contrast, animals in the Hem-Clob group exhibited a more pronounced reduction in body weight after surgery. As in the Hem-Veh group, the weight loss occurred during the first three weeks after surgery and then reached a plateau (Figure 2A), but it was greater than in the vehicle-treated lesioned animals and significantly different from the average body weight of intact controls (Mixed-design ANOVA; Time: *p* < 0.001; Time × Group: *p* < 0.001; Figure 2A; One-way ANOVA followed by Fisher’s LSD post hoc test, *p* < 0.05, Figure 2B), suggesting an additive effect of clobetasol treatment. This enhanced weight decline may reflect a general toxicity as well as a systemic glucocorticoid effect on metabolism and muscle mass, in line with the known catabolic actions of corticosteroids.

### 3.1. Limited Recovery of Locomotion After Hemisection and Detrimental Effects of Clobetasol

**Figure 3:**
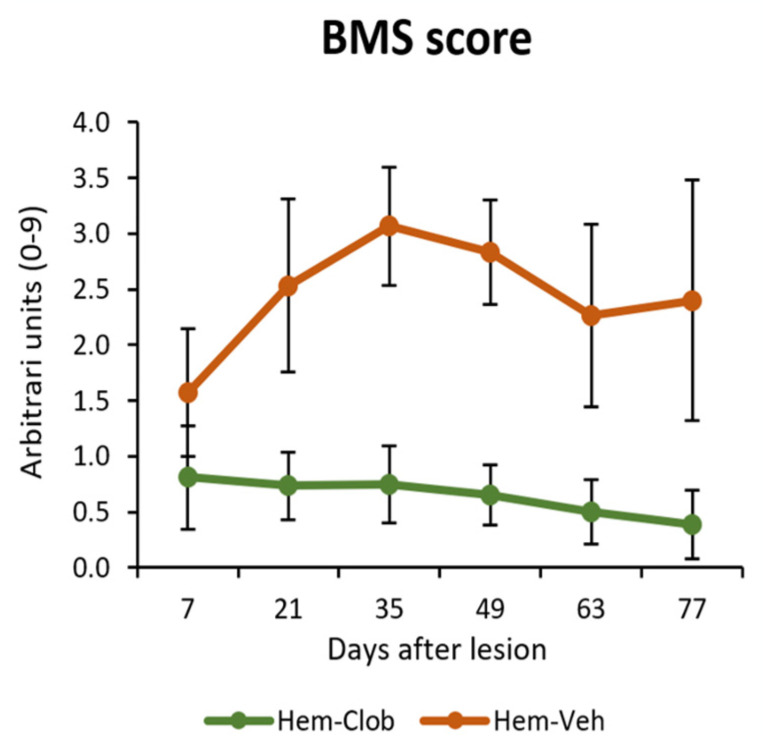
BMS scores in aged mice after spinal cord hemisection. Vehicle-treated animals showed an apparent spontaneous recovery over the first five weeks, reaching values of ~3, whereas clobetasol-treated mice remained at low scores (~0.5–1) throughout the 11 weeks.

As shown in Figure 3, following spinal cord hemisection, aged mice treated with vehicle (Hem-Veh) exhibited a partial but detectable spontaneous recovery of locomotor function. Their BMS scores progressively increased over the first five weeks post lesion, and thereafter stabilized without further improvement. In contrast, Hem-Clob animals showed persistently low scores (~0.5–1) throughout the entire 11-week follow-up period, indicating a lack of recovery. Statistical analysis confirmed a significant treatment effect (Mixed-design ANOVA, Groups: *p* < 0.05; Figure 3).

**Figure 4:**
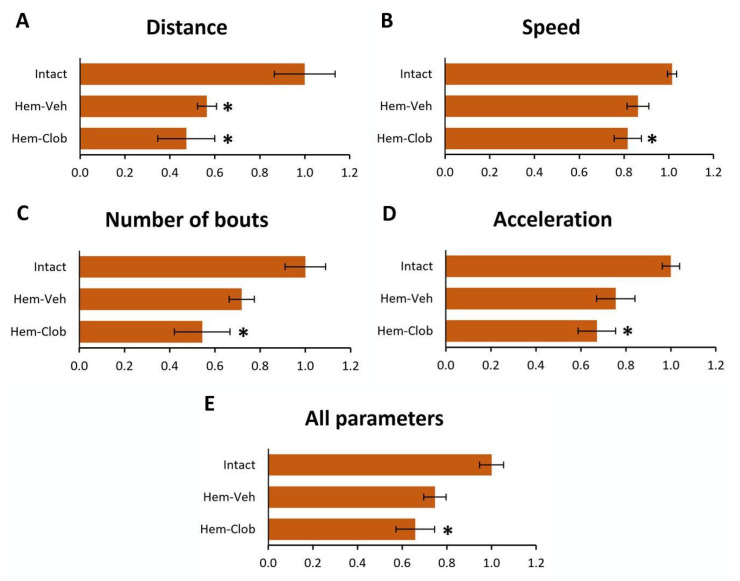
Open field analysis. Vehicle-treated lesioned animals displayed reduced locomotor performance compared with intact controls but retained some residual activity. In contrast, clobetasol-treated mice exhibited significantly worse outcomes across all parameters measured, including distance traveled (**A**), speed (**B**), number of bouts (**C**), and acceleration (**D**). The composite locomotor index (**E**) confirmed a global impairment in the clobetasol group. The asterisk indicates significant difference (*p* < 0.05) from the Intact group.

Open field analysis provided converging evidence (Figure 4). Hem-Veh animals displayed reduced locomotor performance compared with intact controls. In particular, the total distance traveled during the test was significantly reduced (One-way ANOVA followed by Fisher’s LSD post hoc test, *p* < 0.05; Figure 4A), but the other parameters, including speed, acceleration, and number of locomotor bouts appeared to be partially reduced, but the reduction was not statistically significant (One-way ANOVA followed by Fisher’s LSD post hoc test, *p* > 0.05; Figure 4B–D). 

In contrast, Hem-Clob mice performed significantly worse in every parameter assessed. All parameters were markedly reduced relative to intact mice (Figure 4A–D). When all parameters were integrated into a composite locomotor index (Figure 4E), clobetasol-treated mice consistently scored the lowest, confirming a global impairment of spontaneous activity.

Taken together, these findings demonstrate that in aged mice, spinal cord hemisection leads to a modest spontaneous recovery of locomotor function that is evident in both BMS scores and open field performance. Importantly, this recovery was completely absent in clobetasol-treated mice, which not only failed to improve but also exhibited a significant worsening of locomotor parameters.

These results indicate that clobetasol administration under these conditions does not promote plasticity or functional restoration but instead aggravates motor deficits after spinal cord injury in aged animals.

### 3.2. Effects of Hemisection and Clobetasol on Synaptic Plasticity and Neuroinflammation

**Figure 5:**
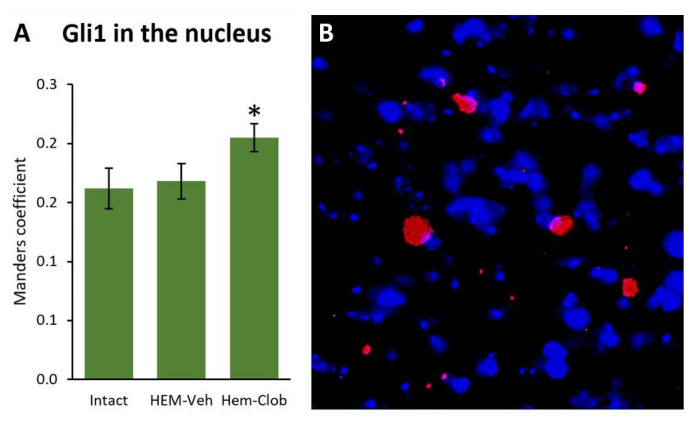
Gli1 and DAPI colocalization in the spinal cord. Lesioned mice treated with clobetasol showed a marked increase (**A**) of colocalization between Gli1 immunostaining (red in (**B**)) with nuclear DAPI staining (blue in (**B**)) compared to both intact and vehicle-treated animals (**A**). The asterisk indicates significant difference (*p* < 0.05) from the Intact group.

To further investigate the cellular correlates of the behavioral findings, synaptic integrity and microglia/macrophage reaction have been examined in lumbar spinal cord sections collected 11 weeks after injury. Moreover, the nuclear localization of Gli1, which is the downstream effector of the canonical Shh pathway activation, has been evaluated by measuring the localization of Gli1 in the nuclei. The results have shown that the Gli1/DAPI colocalization is significantly increased in the spinal cords of animals treated with clobetasol, thus confirming that the drug was able to enter the spinal cord and activate the pathway (One-way ANOVA followed by Fisher’s LSD post hoc test, *p* < 0.05, Figure 5A).

**Figure 6:**
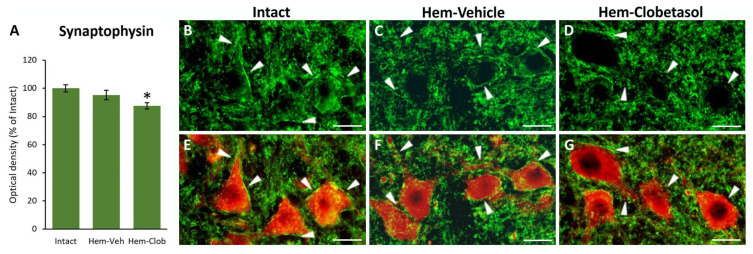
Synaptophysin (green) and ChAT (red) immunostaining in the spinal cord. Lesioned mice treated with clobetasol (**D**,**G**) showed a marked reduction in synaptophysin-positive puncta compared with intact (**B**,**E**) or vehicle-treated (**C**,**F**) animals. White arrowheads indicate synaptic densities on the motoneuron surface. Quantitative analysis confirmed a significant decrease in synaptophysin immunoreactivity after clobetasol treatment, indicating reduced synaptic density (**A**). The asterisk indicates significant difference (*p* < 0.05) from the Intact group. Scale bar: 30 μm.

As shown in Figure 6, immunostaining for synaptophysin revealed a marked reduction in presynaptic puncta in Hem-Clob animals (arrowheads in D or G) compared with intact controls (arrowheads in B, or E), as well as with Hem-Veh group (arrowheads in C or F). Quantitative analysis confirmed that synaptophysin immunoreactivity was significantly decreased in the Hem-Clob group (One-way ANOVA followed by Fisher’s LSD post hoc test, *p* < 0.05, Figure 6A), indicating a loss of synaptic density associated with clobetasol treatment. These findings are consistent with the impaired motor performance observed in behavioral tests, suggesting that clobetasol not only prevents functional recovery but also negatively impacts synaptic connectivity in the injured spinal cord.

**Figure 7:**
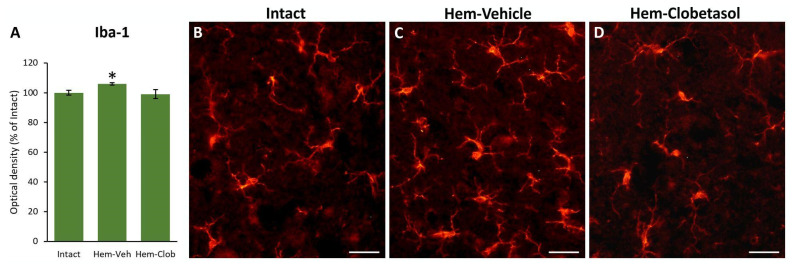
Iba1 immunostaining in the spinal cord. Vehicle-treated lesioned mice (**A**,**C**) exhibited an increase in Iba1 expression compared to intact controls (**A**,**B**), reflecting microglia/macrophage reaction after injury. In contrast, clobetasol-treated animals (**A**,**D**) did not display a significant elevation in Iba1 levels, suggesting that clobetasol suppressed microglial reactivity. The asterisk indicates significant difference (*p* < 0.05) from the Intact group. Scale bar: 30 μm.

Figure 7 shows microglia/macrophage reaction using Iba1 immunostaining. Vehicle-treated lesioned animals displayed a significant increase in Iba1+ cells compared with intact controls (One-way ANOVA followed by Fisher’s LSD post hoc test, *p* < 0.05, Figure 7A; compare panels B and C), reflecting a microglial reactivity after SCI. In contrast, clobetasol-treated animals did not show any significant increase in Iba1 expression, suggesting that clobetasol blunted the microglial response to injury (Figure 7D). This effect may be attributable, at least partially, to the potent glucocorticoid activity of clobetasol, which is known to exert antinflammatory activity.

Together, these histological findings indicate that in aged mice, clobetasol administration exerts two opposing effects: while it suppresses microglial reaction, it simultaneously reduces synaptic density, resulting in an overall worsening of locomotor function.

## 4. Discussion

This study examined the effects of clobetasol treatment in aged mice after spinal cord hemisection, focusing on both motor activity and histological correlates. Behavioral data showed that vehicle-treated aging animals displayed a modest spontaneous recovery in locomotor function, whereas clobetasol treatment not only failed to improve outcomes but appeared to impede recovery. Histologically, synaptic densities were decreased in clobetasol-treated animals, while microglia/macrophage reaction was increased in injured, untreated mice but not in clobetasol-treated ones. These results may appear in contrast with previous studies using selective Shh agonists that consistently showed enhanced progenitor proliferation, synaptic preservation, and improved motor function [30,31]. Two critical differences may account for this discrepancy: first, our use of aged mice, in which intrinsic growth capacity and environmental permissiveness are already limited, and second, the pharmacological profile of clobetasol, which combines Smoothened agonism with potent glucocorticoid receptor activation. The latter likely resulted in excessive immunosuppression and downregulation of synaptic proteins, thereby negating any potential pro-regenerative effects of Shh/Smo stimulation, which is proven by the increased translocation of Gli1 to the nucleus. These findings underscore that therapeutic strategies effective in young animals could not be assumed to translate directly to older subjects.

The present study aligns with this landscape, showing that untreated mice exhibited only partial spontaneous recovery, with BMS scores plateauing at a relatively low level, and open field tests indicating limited ambulatory and exploratory function. The inability to mount robust recovery in aging mice likely reflects reduced intrinsic growth capacity of neurons (e.g., reduced mTOR, PTEN/SOCS3 axis inefficiency), decreased synaptic maintenance and plasticity, and an extrinsic environment that becomes more inhibitory: increased myelin inhibitors, impairment of glial scar resolution, reduced angiogenic response, and diminished immune regulation [13,14,15,16,17,18,19,20,21,22].

Clobetasol is a synthetic glucocorticoid with known ability to agonize Smo, thereby engaging the Shh pathway, at least under some conditions, which has been shown to promote neurogenesis, oligodendrocyte precursor differentiation, remyelination, synaptic maintenance, and reductions in apoptosis in diverse cell models or CNS injury models [39,40,41,46]. Although some of these actions can be attributed to the glucocorticoid activity, it has been proven that the majority of the cited effects are linked to the Smo activity, as demonstrated by the comparison with other Smo agonists and antagonists, as well as with other glucocorticoids with or without Smo activity [46].

The hypothesis of the present work was that in aged mice, clobetasol might compensate for age-related declines in endogenous Shh/Smo signaling and thus facilitate plasticity after SCI. Instead, the results indicate the opposite. As discussed in earlier studies, Shh/Smo signaling is essential for preserving plasticity in neural precursor cells, for stimulating remyelination, for promoting synapse formation, etc. For example, Wang and collaborators [35] showed that Smo deletion in neural stem cells accelerates age-associated neurogenic decline and impairs functional recovery after stroke. Other works from our laboratory has shown that Shh pathway is crucial in the spinal cord plasticity and that clobetasol can easily engage the Smo receptor in the CNS and rescue Shh signaling in models of motoneuron loss, improve synaptic plasticity, and reduce inflammatory markers [40,46,47,48]. Numerous other studies in the literature have highlighted an active role of the Shh/Smo pathway in promoting regeneration and functional recovery in models of spinal cord and peripheral nerve injury. For instance, Shh can modulate inflammatory response, attenuate blood–spinal cord barrier permeability, promote proliferation and reduce apoptosis of nerve cells, and improve functional recovery after spinal cord injury [33,49,50,51].

However, the bulk of that evidence comes from younger or middle-aged animals, and the present results suggest that in advanced age and in the context of SCI, the benefit/risk balance of clobetasol shifts, possibly with glucocorticoid effects overwhelming Smo-mediated repair. The glucocorticoid receptor-mediated effects of clobetasol may dominate over its Smo agonist effects. Although the activation of the Shh pathway can provide beneficial effects, glucocorticoids are well-known to exert strong immunosuppressive effects, and the use of these drugs in SCI can produce unexpected outcomes, thus providing controversial evidence about the helpfulness of these drugs in SCI, also because of their systemic adverse effects [52,53,54]. In aged CNS, where the regenerative machinery is already compromised, suppression of inflammation (which in acute phases may carry beneficial clean-up and remodeling roles) or dampening of synaptic gene expression may lead to net negative outcomes.

The presence of systemic adverse effects can also be supposed in the present study, given the significant reduction in body weight in clobetasol-treated SCI animals. Moreover, histological data support the hypothesis of deleterious effects of clobetasol on spinal cord plastic changes. A decrease in synaptophysin immunostaining suggests reduced presynaptic boutons and fewer functional synapses in the clobetasol-treated group. This correlates with worse locomotor behavior, which depends on preserved synaptic integrity between descending fibers, propriospinal networks, interneurons, and motoneurons. In SCI, preservation of synaptic contacts is critical for effective signal transmission; loss of synapses may reflect axonal dieback, failure of sprouting, or lack of reinnervation. The present results suggest that clobetasol may contribute to synaptic decline rather than preservation in aged SCI, as demonstrated in several experimental models where corticosteroids or chronic stress caused alterations in synaptic plasticity [55,56,57,58].

On the other hand, Iba1 staining showed an increased microglia/macrophage reaction in injured, untreated animals, which is typical after a CNS lesion [3,5,6]; however, in clobetasol-treated mice, this elevation was blunted. It is possible that this suppression of microglial reaction reduced harmful chronic inflammation, but also curtailed beneficial microglial functions (e.g., phagocytosis of debris, release of growth-promoting cytokines, synaptic remodeling) [59,60], necessary especially in aged tissue.

Other mechanisms underlying the detrimental effects of clobetasol can include metabolic imbalance: aged neurons have reduced mitochondrial reserve, increased oxidative stress, and protein homeostasis stress. As seen in recent studies, glucocorticoids can exacerbate metabolic stress, interfere with mitochondrial biogenesis or autophagy, further impairing neuronal/synaptic maintenance [55,61].

## 5. Conclusions

In contrast with previous studies from this laboratory, employing younger (3–4 months old) animals and different models, the present study suggests that in aged mice after spinal cord hemisection, spontaneous plasticity is limited and clobetasol treatment fails to improve locomotor recovery, and indeed appears to exacerbate functional deficits. Histologically, there is a reduction in synaptic density (synaptophysin) and suppression of microglia/macrophage reaction (Iba1) in clobetasol-treated animals. Together, these results suggest that in aged CNS, the balance between beneficial regenerative signals (such as those mediated by Shh/Smo) and inhibitory or suppressive effects (largely glucocorticoid receptor-mediated) is shifted unfavorably by clobetasol under our experimental conditions.

These findings have important implications for therapeutic strategies targeting plasticity and regeneration in spinal cord injury, especially in older individuals. More specifically, the data argue for exploring selective Smo agonists devoid of glucocorticoid receptor activation and focusing also on combining treatments that can overcome the multiple age-related barriers to regeneration. Ultimately, successful translation to human SCI will demand interventions that restore synaptic integrity, support appropriate immune responses, and engage intrinsic growth programs, all while avoiding deleterious suppression of plasticity in aging.

## Figures and Tables

**Table 1:** BMS scoring criteria (from Basso et al., 2006, [44]).

Score	Description of Motor Activity
0	No ankle movement
1	Slight ankle movement
2	Extensive ankle movement
3	Plantar placing of the paw with or without weight support -OR- Occasional, frequent or consistent dorsal stepping but no plantar stepping
4	Occasional plantar stepping
5	Frequent or consistent plantar stepping, no coordination -OR- Frequent or consistent plantar stepping, some coordination, paws rotated at initial contact and lift off
6	Frequent or consistent plantar stepping, some coordination, paws parallel at initial contact -OR- Frequent or consistent plantar stepping, mostly coordinated, paws rotated at initial contact and lift off
7	Frequent or consistent plantar stepping, mostly coordinated, paws parallel at initial contact and rotated at lift off -OR- Frequent or consistent plantar stepping, mostly coordinated, paws parallel at initial contact and lift off, and severe trunk instability
8	Frequent or consistent plantar stepping, mostly coordinated, paws parallel at initial contact and lift off, and mild trunk instability -OR- Frequent or consistent plantar stepping, mostly coordinated, paws parallel at initial contact and lift off, and normal trunk stability and tail down or up and down
9	Frequent or consistent plantar stepping, mostly coordinated, paws parallel at initial contact and lift off, and normal trunk stability and tail always up

## Data Availability

The data presented in this study are available on request from the corresponding author.

## Abbreviations

The following abbreviations are used in this manuscript:

SCI	Spinal Cord Injury
CNS	Central Nervous System
PTEN	Phosphatase and TENsin homolog
mTOR	Mammalian Target of Rapamycin
BMS	Basso Mouse Scale
OCT	Optimal Cutting Temperature
Shh	Sonic hedgehog
Smo	Smoothened
ChAT	Choline Acetyltransferase
